# Efficacy and Safety of Adjunctive Recombinant Human Interleukin-2 for Patients with Pulmonary Tuberculosis: A Meta-Analysis

**DOI:** 10.1155/2022/5071816

**Published:** 2022-11-23

**Authors:** Lina Sheng, Xiaofei Li, Fangbin Weng, Shuang Wu, Yongxin Chen, Lianqing Lou

**Affiliations:** Department of Infectious Diseases, Yiwu Central Hospital, Jinhua, Zhejiang 322000, China

## Abstract

**Background:**

The results of previous clinical trials evaluating the efficacy and safety of recombinant human interleukin-2 (rhuIL-2) for adult patients with pulmonary tuberculosis showed inconsistent results. Accordingly, a comprehensive systematic review and meta-analysis was performed.

**Methods:**

Relevant randomized controlled trials (RCTs) were retrieved by searching the PubMed, Embase, Cochrane's Library, Web of Science, Wanfang, and CNKI databases. A random-effects model was used to combine the results.

**Results:**

18 RCTs with 2630 patients were included in this meta-analysis. Pooled results showed that adjunctive rhuIL-2 significantly increased the odds of sputum culture conversion to negative (risk ratio [RR]: 1.27, 95% CI: 1.09 to 1.47, *p*=0.002, *I*^2^ = 80%), sputum smear conversion to negative (RR: 1.35, 95% CI: 1.17 to 1.57, *p* < 0.001, *I*^2^ = 83%), radiographic focus absorption (RR: 1.17, 95% CI: 1.06 to 1.30, *p*=0.002, *I*^2^ = 72), and cavity closure (RR: 1.24, 95% CI: 1.09 to 1.40, *p* < 0.001, *I*^2^ = 23). The use of rhuIL-2 was not related to any severe adverse events which led to discontinuation of the treatment. Results showed that rhuIL-2 was related to an increased risk of fever (RR: 2.46, 95% CI: 1.29 to 4.70, *p*=0.006, *I*^2^ = 0%). The incidence of other adverse events, such as musculoskeletal pain, hepatic injury, and renal toxicity, was not significantly different between groups (*p* all >0.05).

**Conclusions:**

rhuIL-2 is an effective adjunctive immunotherapy for patients with pulmonary tuberculosis.

## 1. Introduction

Tuberculosis (TB) is a major infectious disease and a serious public health problem worldwide, which is caused by the infection of *Mycobacterium tuberculosis* (*Mtb*) [1, 2]. As of 2020, the World Health Organization (WHO) has proximately estimated 9.9 million new cases and 1.28 million deaths of TB in the global population [3]. Currently, the cornerstone for the treatment of TB is the standard chemotherapy [4, 5]. However, some inevitable problems during chemotherapy still exist, including a long treatment course, severe adverse effects, poor compliance, and resistance to multiple drugs [6, 7]. Accordingly, efforts to develop novel adjunctive therapy for patients with TB are still of great significance in clinical medicine and public health.

Accumulating evidence suggests that a host's ability to recognize, respond to, and regulate MTB determines the occurrence, development, and outcome of TB [8–10]. Because activated macrophages and specific T cells work in concert to protect the host against TB through the production and interaction of innate immune cells, treatments that enhance protective immunity or regulate adaptive immunity against TB are potential adjuvants for patients with advanced disease [11, 12]. It has been confirmed in preclinical studies that immune activation and regulation are both mediated by interleukin-2, a cytokine associated with Th1-type immune responses [13, 14]. Besides, pilot studies also showed that IL-2 could cause differential gene expression in peripheral blood mononuclear cells (PBMCs) stimulated by *Mtb* [15] and enhance the proliferation and transformation of CD4^+^ T cells and NK cells [16], which might collectively enhance the anti-TB efficacy of the standard chemotherapy. However, previous clinical trials evaluating the influence of recombinant human interleukin-2 (rhuIL-2) as adjuvant to chemotherapy in adult patients with pulmonary TB showed inconsistent results [16–33]. Besides, it remains largely unknown whether the potential efficacy and safety of adjunctive rhuIL-2 treatment are similar in patients with drug-susceptible and multidrug-resistant TB (MDR-TB) and in patients with newly diagnosed and recurrent TB. Therefore, we performed a meta-analysis to comprehensively summarize the efficacy and safety of adjunctive rhuIL-2 treatment on the basis of standard chemotherapy in adults with pulmonary TB.

## 2. Methods

The PRISMA (Preferred Reporting Items for Systematic Reviews and Meta-Analyses) statement [34, 35] and the Cochrane Handbook guidelines [36] were followed during the design and implementation of the study.

### 2.1. Search Strategy

The Medline (PubMed), Embase (Ovid), CENTER (Cochrane Library), Web of Science, Wanfang, and China National Knowledge Infrastructure (CNKI) databases were searched for relevant studies with a combined strategy of [1] “interleukin-2” or “IL-2” or “recombinant human IL-2” or “rhuIL-2”; [2] “tuberculosis” or “*Mycobacterium tuberculosis* infection” or “tuberculous lesion” or “tuberculoses” or “Kochs Disease”; and [3] “random” or “randomized” or “randomized” or “randomly” or “RCT” or “placebo.” Only studies including human subjects were considered. The references of related reviews and original articles were also searched for relevant studies. The final database search was conducted on August 29, 2022.

### 2.2. Study Selection

Studies that fulfilled the following criteria were included: [1] full-length articles published in English or Chinese; [2] designed as parallel-group RCTs; [3] adult patients who were diagnosed with HIV-seronegative pulmonary TB and randomly allocated to a treatment group with adjunctive rhuIL-2 and a control group without rhuIL-2 on the basis of standard chemotherapy for TB; and [4] reported at least one of the following efficacy outcomes, including the proportion of patients with sputum culture conversion to negative, the proportion of patients with sputum smear conversion to negative, the proportion of patients with radiographic focus absorption, and the proportion of patients with radiographic cavity closure. Radiographic changes of the pulmonary TB focuses were rated to the following four grades as previously described: marked absorption (meaning significant improvement of more than half of initial abnormalities), moderate absorption (meaning definite improvement better than initial abnormalities but less than a half), no changes (no certain difference in films compared with original lesion), and deterioration (being worse than initial abnormalities or spreading to another area) [37]. The combined proportions of patients with marked and moderate absorption were considered as those with focus absorption. No restriction was applied to the dosage, route, and duration of rhuIL-2 treatment [37]. Nonrandomized studies, studies including patients without pulmonary TB, or studies that did not report the outcomes of interest were excluded. For studies with overlapped patient population, the one with the largest sample size was included for the meta-analysis.

### 2.3. Data Extraction and Quality Assessment

Database searches, data extraction, and quality evaluation were conducted by two independent authors. If disagreement occurred, it was resolved by discussion with the corresponding author. We extracted data regarding study information (first author, publication year, and study country), study design (blind or open-label), and patient information (number of patients, mean age, sex, MDR or drug-susceptible TB, and newly diagnosed or recurrent pulmonary TB), background treatments, dosages, routes, and the duration of rhuIL-2 treatment, regimens of controls, and follow-up duration). Quality evaluation was achieved using the Cochrane's Risk of Bias Tool [36] according to the following aspects: [1] random sequence generation, [2] allocation concealment, [3] blinding of participants and personnel, [4] blinding of outcome assessors, [5] incomplete outcome data, [6] selective outcome reporting, and [7] other potential bias.

### 2.4. Statistical Analysis

The methodology of statistics is generally considered with the previous published meta-analysis involving RCTs [38]. The influence of adjunctive rhuIL-2 on the proportion of patients who achieved the efficacy outcomes were presented as risk ratios (RRs) and the corresponding 95% confidence intervals (CIs). Besides, the influence of rhuIL-2 on the risks of common adverse events, including fever, musculoskeletal pain, hepatic injury, and renal toxicity, were also summarized as RRs and 95% CIs. We used the Cochrane's *Q*-test to detect the heterogeneity [39]. The *I*^2^ statistic was also calculated, and an *I*^2^>50% reflected significant heterogeneity [40]. Pooled analyses were calculated using a random-effects model because this method incorporates the influence of potential heterogeneity and provides a more generalized result [36]. Sensitivity analysis by exclusion of one study at a time was used to evaluate the influence of each study on the pooled results of the meta-analysis [36]. Additionally, subgroup analyses were performed to evaluate whether the results were similar in patients with MDR-TB and drug-susceptible TB and in patients with newly diagnosed and recurrent TB. Meta-regression analyses were performed to evaluate the possible influence of patient and treatment characteristics on the efficacy outcomes, including the number of patients, the mean age, the mean daily dose, routes, and the duration of rhuIL-2 treatment. Publication bias was evaluated by visual inspection of funnel plots and Egger's regression asymmetry test [41]. Differences with *p* < 0.05 were considered statistically significant. The RevMan (Version 5.1; Cochrane, Oxford, UK) and Stata software (version 12.0; Stata Corporation, College Station, TX) were used for the statistical analyses.

## 3. Results

### 3.1. Search Results

**Figure 1:**
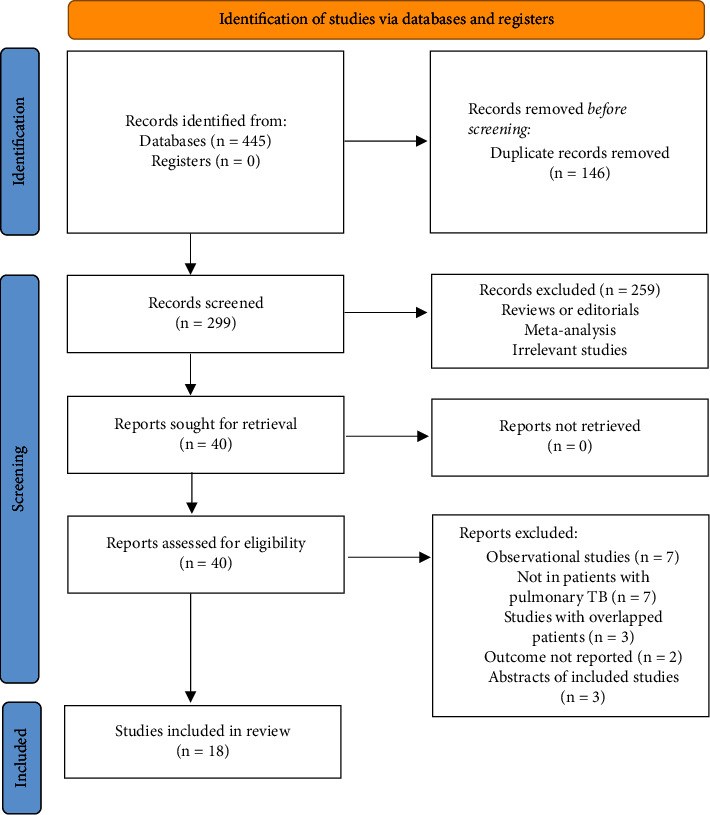
Flowchart of literature search.

The process of database search and study identification is illustrated in Figure 1. Briefly, 445 articles were obtained through the database search, and 299 were retrieved after exclusion of duplicate records. Among them, 259 articles were subsequently excluded based on titles and abstracts primarily because these studies were irrelevant to the aim of the meta-analysis. Of the 40 articles that underwent full-text review, 22 were further excluded for the reasons presented in Figure 1. Finally, 18 RCTs [16–33] were included.

### 3.2. Study Characteristics and Data Quality

Table 1 shows the characteristics of the included studies. Overall, 18 RCTs [16–33] with 2630 adult patients with pulmonary TB were included in this meta-analysis. According to the treatment, 1332 patients were allocated to adjunctive rhuIL-2, and 1298 were allocated to standard chemotherapy alone. These studies were published between 1997 and 2022 and mostly performed in China expect for two studies, which included patients from South Africa [17] and Uganda [18]. The mean age of the patients varied between 35 and 57 years, and all the patients received standard chemotherapy for TB. Six of the studies included patients with MDR-TB [17, 20, 22, 28–30], and another six RCTs included patients with drug-susceptible TB [18, 24–26, 32, 33]. Among the remaining six studies [16, 19, 21, 23, 27, 31], one study included patients with MDR-TB or drug-susceptible TB [16], while the other five studies did not report the TB drug sensitivity status of the patients [19, 21, 23, 27, 31]. In addition, patients with newly diagnosed TB were included in six studies [18, 24–26, 32, 33], patients with recurrent TB were included in ten studies [16, 17, 19, 20, 22, 23, 27–29, 31], while the remaining two studies included patients with newly diagnosed or recurrent TB [21, 30]. The dosages of rhuIL-2 varied between 200,000 IU and 1000,000 IU per day, and the treatment duration varied from 1 to 4 months. The follow-up duration was 1 ∼ 24 months. The detailed quality evaluation of the included RCTs via the Cochrane risk of bias tool is shown in Table 2. Two of the included studies were double-blinded [17, 18]. The methods of random sequence generation were reported in seven studies [17, 18, 20, 27, 28, 32, 33], and the details of allocation concealment were reported in two studies [17, 18].

### 3.3. Microbiologic Outcomes

**Figure 2:**
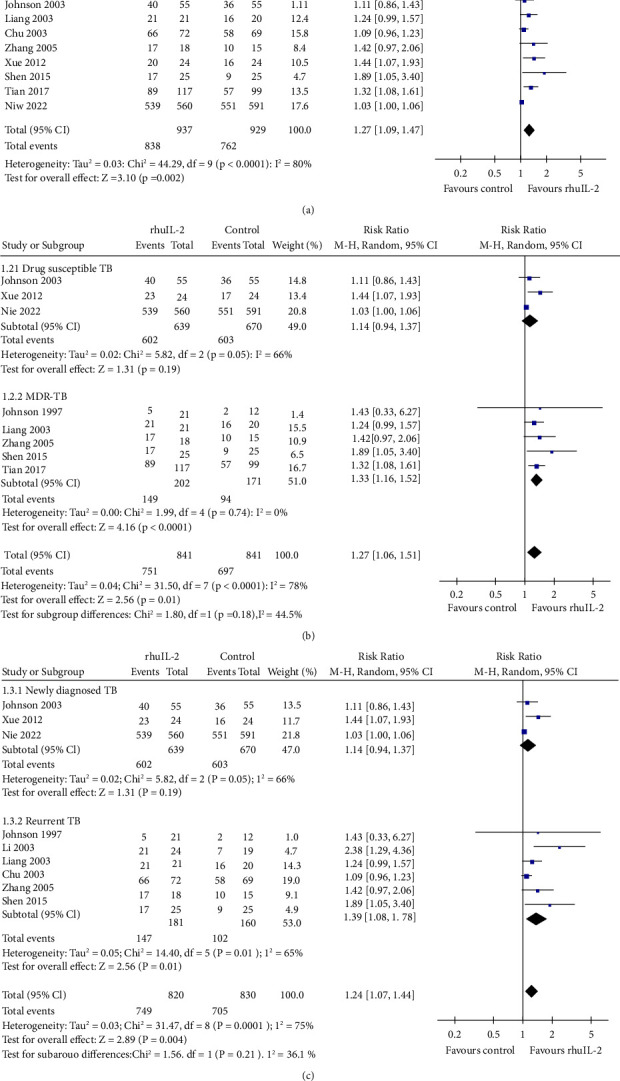
Forest plots for the meta-analysis of the effect of rhuIL-2 on the proportion of patients with sputum culture conversion to negative; (a), forest plots for the overall meta-analysis; (b), forest plots for the subgroup analysis according to the drug sensitivity of TB; and (c), forest plots for the subgroup analysis in newly diagnosed and recurrent TB.

**Figure 3:**
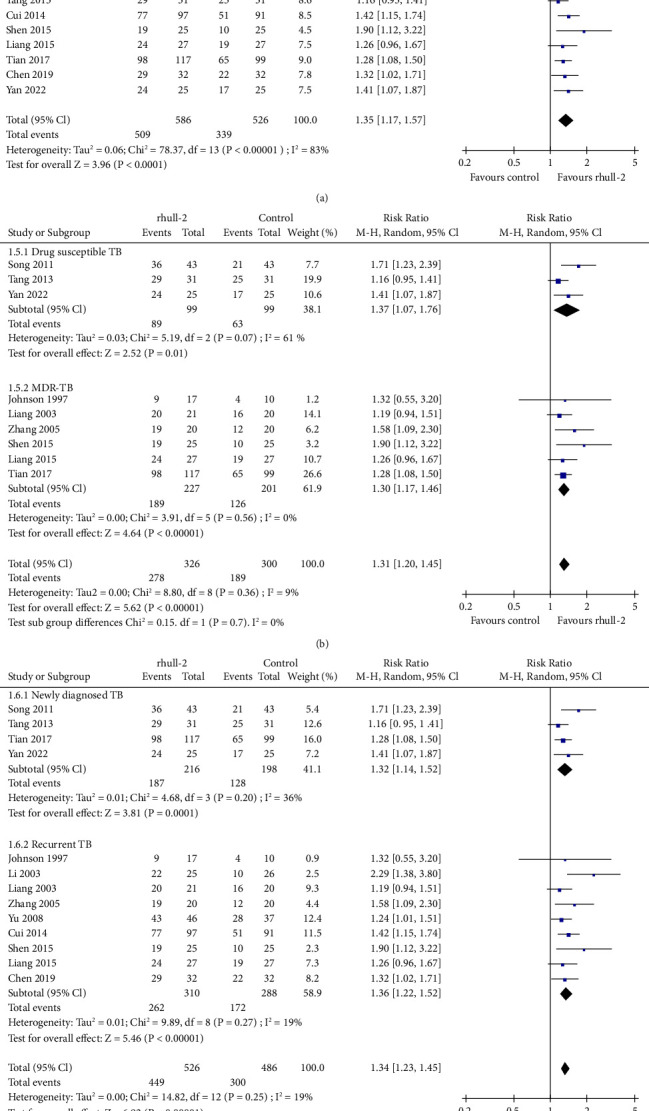
Forest plots for the meta-analysis of the effect of rhuIL-2 on the proportion of patients with sputum smear conversion to negative; (a), forest plots for the overall meta-analysis; (b), forest plots for the subgroup analysis according to the drug sensitivity of TB; and (c), forest plots for the subgroup analysis in newly diagnosed and recurrent TB.

Pooled results of 10 RCTs [16–20, 22, 25, 29, 30, 32] showed that adjunctive rhuIL-2 significantly increased the proportion of patients who achieved sputum culture conversion to negative (RR: 1.27, 95% CI: 1.09 to 1.47, *p*=0.002, *I*^2^ = 80%; Figure 2(a)). Sensitivity analysis by excluding one study at a time showed consistent result (RR: 1.22 ∼ 1.33, *p* all <0.05). Further subgroup analyses showed that adjunctive rhuIL-2 significantly increased the odds of sputum culture conversion to negative in patients with MDR-TB (RR: 1.33, *p* < 0.001) but not in those with drug-susceptible TB (RR: 1.14, *p*=0.19; Figure 2(b)). Besides, subgroup analyses also showed that adjunctive rhuIL-2 significantly increased the odds of sputum culture conversion to negative in patients with recurrent TB (RR: 1.39, *p*=0.01) but not in the patients with newly diagnosed TB (RR: 1.14, *p*=0.19; Figure 2(c)). Further meta-analysis with 14 RCTs [17, 19–24, 26–31, 33] showed that adjunctive rhuIL-2 significantly increased the proportion of patients who achieved sputum smear conversion to negative (RR: 1.35, 95% CI: 1.17 to 1.57, *p* < 0.001, *I*^2^ = 83%; Figure 3(a)). Sensitivity analysis by excluding one study at a time did not significantly change the result (RR: 1.32 ∼ 1.38, *p* all <0.05). Further subgroup analyses showed consistent results in patients with drug-susceptible TB (RR: 1.37, *p*=0.01) and MDR-TB (RR: 1.37, *p* < 0.001; Figure 3(b)) and in patients with newly diagnosed (RR: 1.32, *p* < 0.001) and recurrent TB (RR: 1.36, *p* < 0.001; Figure 3(c)). Further univariate meta-regression analyses did not show that characteristics including the number of patients, the mean age, the mean daily dose, routes, or the duration of rhuIL-2 treatment had a significant influence on the effect of rhuIL-2 for the above microbiologic outcomes (*p* all >0.05, Table 3).

### 3.4. Radiographic Outcomes

**Figure 4:**
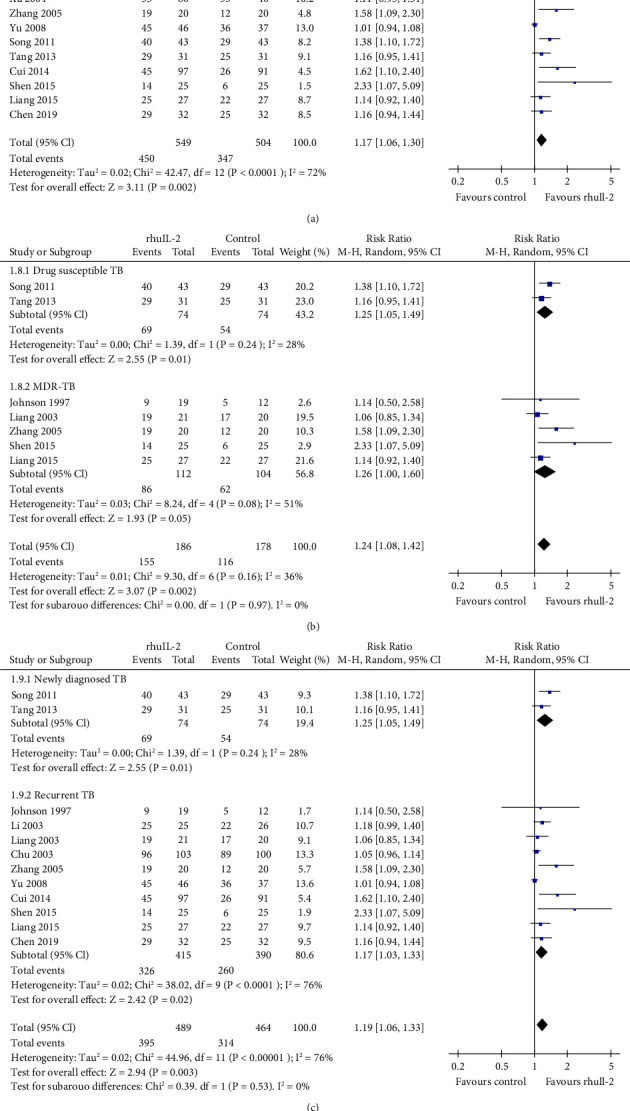
Forest plots for the meta-analysis of the effect of rhuIL-2 on the proportion of patients with radiographic focus absorption; (a), forest plots for the overall meta-analysis; (b), forest plots for the subgroup analysis according to the drug sensitivity of TB; and (c), forest plots for the subgroup analysis in newly diagnosed and recurrent TB.

**Figure 5:**
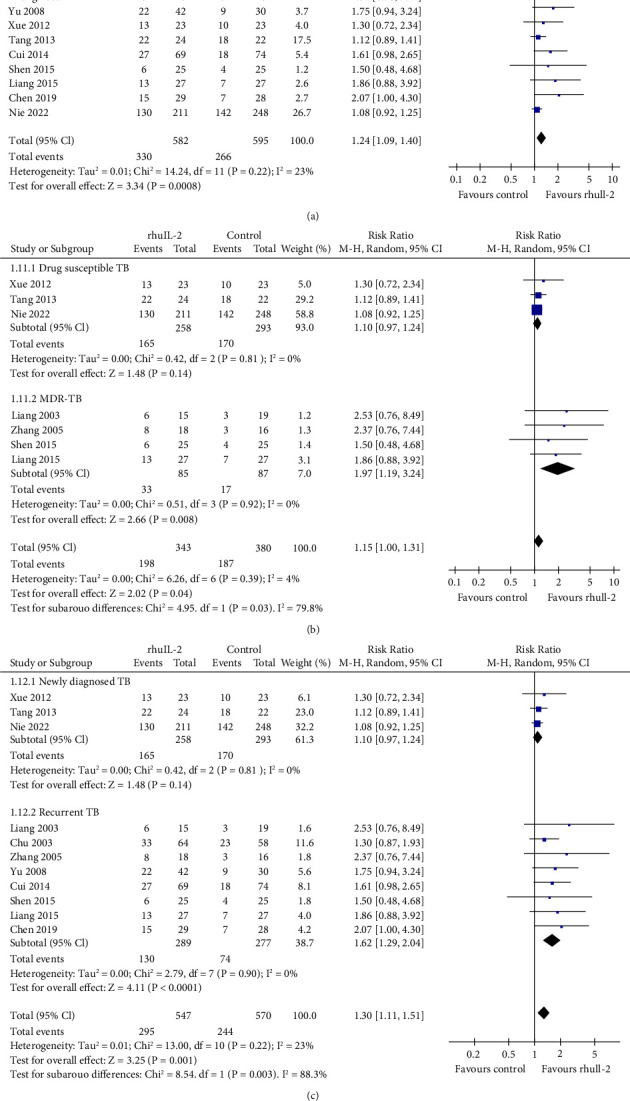
Forest plots for the meta-analysis of the effect of rhuIL-2 on the proportion of patients with radiographic cavity closure; (a), forest plots for the overall meta-analysis; (b), forest plots for the subgroup analysis according to the drug sensitivity of TB; and (c), forest plots for the subgroup analysis in newly diagnosed and recurrent TB.

The eesults of meta-analysis including 13 RCTs [16, 17, 19–24, 26–29, 31] showed that rhuIL-2 significantly increased the proportion of patients who achieved radiographic focus absorption (RR: 1.17, 95% CI: 1.06 to 1.30, *p*=0.002, *I*^2^ = 72; Figure 4(a)). The results were not significantly affected by sensitivity analyses by omitting one study at a time (RR: 1.14 ∼ 1.21, *p* all <0.05). Further subgroup analyses showed consistent results in patients with drug-susceptible TB (RR: 1.25, *p*=0.01) and MDR-TB (RR: 1.26, *p*=0.04; Figure 4(b)) and in patients with newly diagnosed (RR: 1.25, *p*=0.01) and recurrent TB (RR: 1.17, *p*=0.02; Figure 4(c)). In addition, meta-analysis with 12 RCTs [16, 20–23, 25–29, 31, 32] showed that rhuIL-2 significantly increased the proportion of patients who achieved cavity closure (RR: 1.24, 95% CI: 1.09 to 1.40, *p* < 0.001, *I*^2^ = 23; Figure 5(a)), which was unchanged in sensitivity analysis by excluding one study at a time (RR: 1.20 ∼ 1.38, *p* all <0.05). Further subgroup analyses showed that the benefit of adjunctive rhuIL-2 on cavity closure was significant in patients with MDR-TB (RR: 1.97, *p*=0.008) and recurrent TB (RR: 1.62, *p* < 0.001) but not in patients with drug-susceptible TB (RR: 1.10, *p*=0.14; Figure 5(b)) and newly diagnosed TB (RR: 1.10, *p*=0.14; Figure 5(c)). Further univariate meta-regression analyses did not show that characteristics including the number of the patients, the mean age, the mean daily dose, routes, or the duration of rhuIL-2 treatment had a significant influence on the effect of rhuIL-2 for the above radiographic outcomes (*p* all >0.05, Table 3).

### 3.5. Safety Outcomes

**Figure 6:**
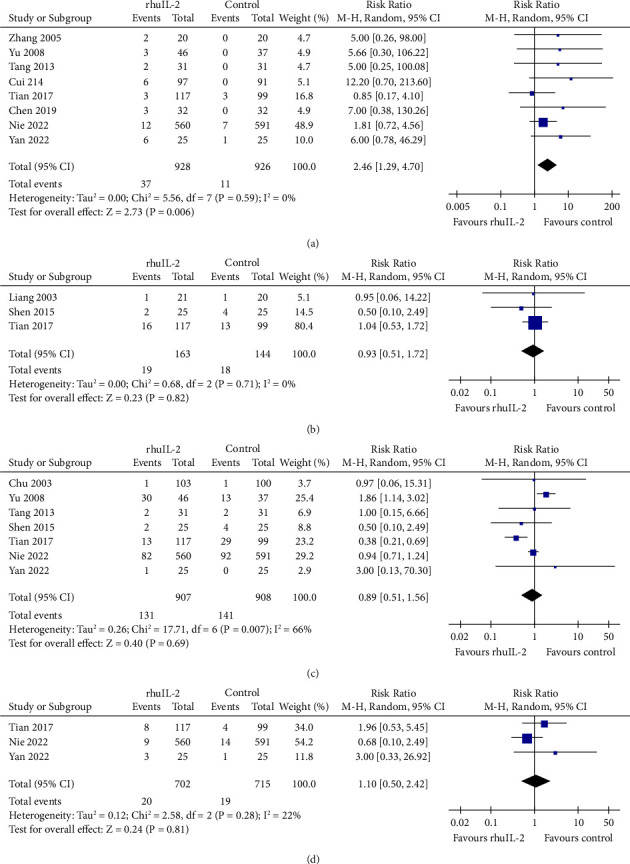
Forest plots for the meta-analysis of the influence of rhuIL-2 on the incidence of adverse events; (a), fever; (b), musculoskeletal pain; (c), hepatic injury; and (d), renal toxicity.

The use of rhuIL-2 was not related to any severe adverse events which led to discontinuation of the treatment in any of the included studies. Results showed that rhuIL-2 was related to an increased risk of fever (RR: 2.46, 95% CI: 1.29 to 4.70, *p*=0.006, *I*^2^ = 0%; Figure 6(a)). The incidence of other adverse events, such as musculoskeletal pain, hepatic injury, and renal toxicity, was not significantly different between groups (Figures 6(b)–6(d), *p* all >0.05).

### 3.6. Publication Bias

**Figure 7:**
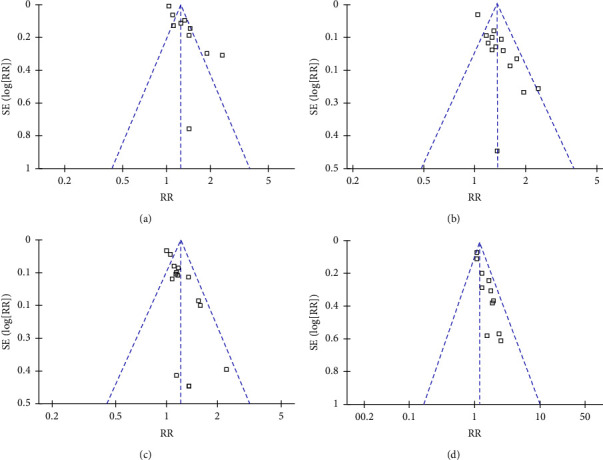
Funnel plots for the publication biases underlying the meta-analyses; (a), funnel plots for the meta-analysis of the effect of rhuIL-2 on the proportion of patients with sputum culture conversion to negative; (b), funnel plots for the meta-analysis of the effect of rhuIL-2 on the proportion of patients with sputum smear conversion to negative; (c), funnel plots for the meta-analysis of the effect of rhuIL-2 on the proportion of patients with radiographic focus absorption; and (d), funnel plots for the meta-analysis of the effect of rhuIL-2 on the proportion of patients with radiographic cavity closure.

The funnel plots for the meta-analyses of the effect of rhuIL-2 on the outcomes of sputum culture conversion, sputum smear conversion, radiographic focus absorption, and cavity closure were symmetrical, suggesting low risk of publication biases (Figures 7(a)–7(d)). Egger's regression tests also suggested low risks of publication biases (*p*=0.17, 0.35, 0.32, and 0.19, respectively). The publication biases underlying the meta-analyses of adverse events were difficult to estimate because only 3 ∼ 8 studies were included for each outcome.

## 4. Discussion

In this systematic review and meta-analysis, we pooled the results of 18 available RCTs, and the results showed that adjunctive treatment with rhuIL-2 on the basis of standard chemotherapy for adult patients with pulmonary TB was associated with improved sputum bacterial elimination and improved radiographic changes. Subgroup analyses showed that the benefits of adjunctive rhuIL-2 remained only among patients with MDR-TB or recurrent TB. Besides, no serious adverse events related to the use of rhuIL-2 were reported. The use of rhuIL-2 may increase the risk of fever, which is generally mild and could be adequately controlled after symptomatic treatment. Taken together, these findings suggest that rhuIL-2 is an effective and safe adjunctive immunotherapy for patients with pulmonary TB who are treated with standard chemotherapy, which is associated with the improved microbiologic and radiographic outcomes.

To the best of our knowledge, only one previous meta-analysis evaluated the potential role of rhuIL-2 as adjunctive immunotherapy in patients with TB [42]. Although the results of the meta-analysis also suggested that rhuIL-2 may improve the sputum TB elimination in patients with TB, only four RCTs were included in the meta-analysis, and only two studies are available for the individual outcomes of sputum culture or smear conversion, which made the results of the meta-analysis less convincing [42]. Besides, the previous meta-analysis failed to show that rhuIL-2 was associated with improved radiographic changes, and the safety of rhuIL-2 was also unable to be determined because of the limited studies available [42]. Our study has a few strengths in methodology as compared to the previous one. First, an extensive literature search was performed in five electronic databases, which retrieved 18 RCTs for the subsequent meta-analysis. The number of the overall included patients was much larger for the current meta-analysis as compared to that of the previous one (2630 versus 656). Second, in addition to the confirmed benefit of rhuIL-2 on sputum TB elimination by pooling 10 and 14 RCTs, respectively, the results of the meta-analysis also indicated that adjunctive rhuIL-2 on the basis of standard chemotherapy may also improve the radiographic changes of patients with TB, including the absorption of the pulmonary focus and the closure of cavities. Moreover, since patients with MDR-TB and recurrent TB have confirmed to be associated with worse prognosis than those with drug-susceptible TB and newly diagnosed TB [43, 44], we performed a subgroup analysis to evaluate if the potential therapeutic efficacy of rhuIL-2 remained for these patients, and the findings confirmed the consistent benefit of rhuIL-2 on microbiologic and radiographic outcomes even in patients with MDR-TB and recurrent TB. Finally, safety outcomes were also evaluated in this meta-analysis, and we found that no severe adverse events related to the use of rhuIL-2 were reported in any of the included studies, and additional treatment with rhuIL-2 only increased the incidence of fever without affecting the hepatic or renal adverse events in patients with pulmonary TB. Taken together, the findings of the current meta-analysis suggested that adjunctive rhuIL-2 is effective and safe in patients with pulmonary TB.

The mechanisms underlying the potential therapeutic efficacy of adjunctive rhuIL-2 for patients with pulmonary TB are likely to be mainly dependent on the role of IL-2 for the restoration and stimulation of the innate immunity of the host against *Mtb*. In order for macrophages to kill mycobacteria, *Mtb*-specific T lymphocytes are essential [45]. It is possible that a dysfunctional cell-mediated immune response to infection with *Mtb* can lead to the progression of the primary infection or to reactivation of TB [45]. Previous studies have shown that IL-2 produced by Th1 cells is essential for the cellular immunity, which however was shown to decrease in patients with TB [46]. Correspondingly, a subsequent study showed that a restored IL-2 level and a significantly elevated IL-2/IFN-*γ* ratio may be a marker for the successful elimination of *Mtb* infection [47], suggesting the possible therapeutic implication of exogenous IL-2 for patients with TB. Consistently, a recent preclinical study in a mouse model of T cell dysfunction by persistent *Mtb* antigen stimulation found a significant decrease in IL-2 production, and the exogenous IL-2 administration restored antigen-specific T cell responses and protective efficacy [48]. Moreover, a recent study suggested that deficiency of IL-2 inducible T cell kinase may impair the early pulmonary protection against *Mtb* infection in mice probably due to the reduced endogenous IL-2 production [49]. The molecular mechanisms underlying the benefits of rhuIL-2 for TB are to be investigated.

The results of the subgroup analyses suggested that in the patients who achieved sputum smear conversion to negative, the rhuIL-2 significantly improved the treatment outcomes in patients with drug-susceptible TB. However, among the proportion of patients who achieved sputum culture conversion to negative, such kind of effects became nonsignificant. Similarly, for the patients with newly diagnosed TB, the usage of rhuIL-2 significantly increased the odds of sputum smear conversion to negative, but the effect became nonsignificant for sputum culture conversion to negative. This may be explained by the low sensitivity of sputum smear for the detection of TB. Indeed, a smear-positive result requires more acid-fast bacilli than sputum culture, and its sensitivity is limited to over 10,000 biological/ml in sputum [50]. Smears for acid-fast bacilli are affected by the specimen material, the patient's intermittent discharge of bacteria, the number of bacteria in the specimen, and many other factors, resulting in low sensitivity [51]. In addition, the results of the subgroup analyses showed that the benefits of rhuIL-2 on sputum TB elimination was mainly driven by studies of patients with MDR-TB and recurrent TB, and the favorable influence of rhuIL-2 on some radiographic change may also be more remarkable in patients with MDR-TB and recurrent TB, such as the closure of pulmonary cavities. These findings highlight an important role of adjunctive rhuIL-2 for patients with MDR-TB and recurrent TB, which is clinically important because anti-TB treatment in these patients is more challenging [52, 53]. The mechanisms are not fully determined. However, it could be hypothesized that the innate immunity of the host against *Mtb* may be impaired more severely in patients with MDR-TB and recurrent TB as compared to those with drug-susceptible and newly diagnosed TB. In fact, a previous study suggested a worse cellular immune function and a lower level of IL-2 in patients with recurrent TB as compared to those of newly diagnosed TB [54]. Future studies are warranted for further investigation.

Our study also has limitations. Firstly, although 18 RCTs were included in the meta-analysis, high-quality large-scale RCTs which evaluate the possible influence of adjunctive rhuIL-2 on clinical outcomes in patients with pulmonary TB remain lacking. Moreover, for the most of the included studies, the follow-up durations are relatively short. Large-scale RCTs with adequate follow-up durations are needed to determine the potential influence of adjunctive rhuIL-2 on the risk of TB recurrence and mortality in these patients, as well as the long-term safety. Besides, the dose, route, and duration of rhuIL-2 administration varied among the included studies. Although results of meta-regression analyses failed to show that difference in these factors have significant influences on the efficacies of rhuIL-2, these results should be interpreted with caution because of the limited available datasets for the analyses. Future studies are needed to determine the optimal regimens for adjunctive rhuIL-2 in patients with pulmonary TB. Finally, only patients with pulmonary TB were included in this meta-analysis. Future studies are needed to determine the possible therapeutic role of adjunctive rhuIL-2 for patients with extra-pulmonary TB.

## 5. Conclusions

In conclusion, the results of the meta-analysis indicate that rhuIL-2 is an effective adjunctive immunotherapy for patients with pulmonary TB, particularly for those with MDR-TB and recurrent TB. Large-scale clinical studies are needed to evaluate the influence of adjunctive rhuIL-2 on long-term clinical prognosis and to determine the optimal regimen of rhuIL-2 for the treatment of patients with pulmonary TB.

## Figures and Tables

**Table 1:** Characteristics of the included RCTs.

Study	Country	Design	No. of patients	Mean age (years)	Men (%)	Drug sensitivity	New or recurrent TB	Background treatment	rhuIL-2 dose and routes	Control	Duration of rhuIL-2 treatment (months)	Follow-up duration (months)
Johnson 1997	South Africa	R, DB, PC	33	36.6	51.4	MDR-TB	Recurrent TB	Standard chemotherapy	225,000 IU i.d. bid, or 450,000 IU i.d. q12 h and resting for 9 days	Placebo	1	1

Li 2003	China	R	51	41.1	70.6	NR	Recurrent TB	Standard chemotherapy	200,000 IU i.d. qd	Blank treatment	2	8

Johnson 2003	Uganda	R, DB, PC	110	35	68.2	Drug-susceptible TB	New TB	Standard short-course chemotherapy	225,000 IU i.d. bid	Placebo	1	2

Liang 2003	China	R	41	40.2	65.9	MDR-TB	Recurrent TB	Standard chemotherapy	200,000 IU i.m. qd for 1 month, resting for 1 month, then repeat the treatment for 1 month	Blank treatment	3	7

Chu 2003	China	R	203	NR	NR	Mixed	Recurrent TB	Standard chemotherapy	200,000 IU i.m. qd for 1 month, resting for 1 month, then repeat the treatment for 1 month	Blank treatment	2	7

Xu 2004	China	R	100	NR	58	NR	Mixed	Standard chemotherapy	200,000 IU s.c. qd for 1 month, resting for 1 month, then repeat the treatment for 1 month	Blank treatment	2	6

Zhang 2005	China	R	40	NR	54.5	MDR-TB	Recurrent TB	Standard chemotherapy	200,000 IU i.d. qd	Blank treatment	3	8

Yu 2008	China	R	83	36	71.1	NR	Recurrent TB	Standard chemotherapy	200,000 IU s.c. qd	Blank treatment	2	8

Song 2011	China	R	86	56.5	NR	Drug-susceptible TB	New TB	Standard chemotherapy	600,000 IU s.c. tiw	Blank treatment	1	1

Xue 2012	China	R	48	NR	NR	Drug-susceptible TB	New TB	Standard chemotherapy	1000,000 IU s.c. tiw	Blank treatment	2	6

Tang 2013	China	R	62	49.5	48.4	Drug-susceptible TB	New TB	Standard chemotherapy	400,000 IU i.d. qd	Blank treatment	3	6

Cui 2014	China	R	188	44.5	40.9	NR	Recurrent TB	Standard chemotherapy	200,000 IU s.c. qd	Blank treatment	2	8

Shen 2015	China	R	50	45.2	82	MDR-TB	Recurrent TB	Standard chemotherapy	500,000 IU i.d. qod for the first, third, fifth, and seventh months	Blank treatment	4	24

Liang 2015	China	R	54	47	51.8	MDR-TB	Recurrent TB	Standard chemotherapy	1000,000 IU s.c. qod	Blank treatment	2	8

Tian 2017	China	R, OL	216	44.1	55.6	MDR-TB	Mixed	Standard chemotherapy	500,000 IU i.d. qod for the first, third, fifth, and seventh months	Blank treatment	4	24

Chen 2019	China	R	64	46.5	62.5	NR	Recurrent TB	Standard chemotherapy	200,000 IU s.c. qod	Blank treatment	3	8

Nie 2022	China	R	1151	42.7	64.3	Drug-susceptible TB	New TB	Standard chemotherapy	400,000 IU i.d. qd	Blank treatment	1	6

Yan 2022	China	R	50	44.7	68	Drug-susceptible TB	New TB	Standard chemotherapy	500,000 IU i.d. qd	Blank treatment	1	6

RCTs, randomized controlled trials; TB, *tuberculosis*; rhuIL-2, recombinant human interleukin-2; R, randomized; DB, double-blind; PC, placebo-controlled; OL, open-label; NR, not reported; MDR-TB, multidrug-resistant *tuberculosis*; i.d., intradermal; bid, twice daily; q12 h, every 12 hours; qd, once daily; i.m., intramuscular; s.c. subcutaneous; tiw, three times a week; qod, every other day.

**Table 2:** Details of quality evaluation via the Cochrane's risk of bias tool.

Study	Random sequence generation	Allocation concealment	Blinding in performance	Blinding in outcome detection	Incomplete outcome data	Reporting bias	Other bias
Johnson 1997	Low risk	Low risk	Low risk	Low risk	Low risk	Low risk	Unclear
Li 2003	Unclear	Unclear	Unclear	Unclear	Low risk	Low risk	Low risk
Johnson 2003	Low risk	Low risk	Low risk	Low risk	Low risk	Low risk	Low risk
Liang 2003	Low risk	Unclear	Unclear	Unclear	Low risk	Low risk	Low risk
Chu 2003	Unclear	Unclear	Unclear	Unclear	Low risk	Low risk	Low risk
Xu 2004	Unclear	Unclear	Unclear	Unclear	Low risk	Low risk	Low risk
Zhang 2005	Unclear	Unclear	Unclear	Unclear	Low risk	Low risk	Low risk
Yu 2008	Unclear	Unclear	Unclear	Unclear	Low risk	Low risk	Low risk
Song 2011	Unclear	Unclear	Unclear	Unclear	Low risk	Low risk	Low risk
Xue 2012	Unclear	Unclear	Unclear	Unclear	Low risk	Low risk	Low risk
Tang 2013	Unclear	Unclear	Unclear	Unclear	Low risk	Low risk	Low risk
Cui 2014	Low risk	Unclear	Unclear	Unclear	Low risk	Low risk	Low risk
Shen 2015	Unclear	Unclear	Unclear	Unclear	Low risk	Low risk	Low risk
Liang 2015	Low risk	Unclear	Unclear	Unclear	Low risk	Low risk	Low risk
Tian 2017	Unclear	Unclear	High risk	High risk	Low risk	Low risk	Low risk
Chen 2019	Unclear	Unclear	Unclear	Unclear	Low risk	Low risk	Low risk
Nie 2022	Low risk	Unclear	Unclear	Unclear	Low risk	Low risk	Low risk
Yan 2022	Low risk	Unclear	Unclear	Unclear	Low risk	Low risk	Low risk

**Table 3:** Univariate meta-regression analyses for the association between patient and treatment characteristics with the efficacy outcomes.

	*RR for sputum culture conversion to negative*		
Covariate	Coefficient	95% CI	*p*
No. of patients	0.047	−0.148 to 0.242	0.67
Mean age (years)	−0.032	−0.099 to 0.035	0.59
rhuIL-2 dose (10^4^ IU/d)	0.0027	−0.0011 to 0.0065	0.18
rhuIL-2 routes	−0.06	−0.17 to 0.05	0.29
Treatment duration (months)	0.18	−0.22 to 0.58	0.49

	*RR for sputum smear conversion to negative*		
Covariate	Coefficient	95% CI	*p*
No. of patients	0.19	−0.04 to 0.42	0.11
Mean age (years)	0.009	−0.017 to 0.035	0.63
rhuIL-2 dose (10^4^ U/d)	−0.013	−0.251 to 0.225	0.92
rhuIL-2 routes	−0.31	−0.97 to 0.35	0.36
Treatment duration (months)	0.33	−0.10 to 0.76	0.18

	*RR for radiographic focus absorption*		
Covariate	Coefficient	95% CI	*p*
No. of patients	0.03	−0.19 to 0.25	0.85
Mean age (years)	−0.044	−0.102 to 0.014	0.13
rhuIL-2 dose (10^4^ IU/d)	−0.052	−0.153 to 0.049	0.36
rhuIL-2 routes	−0.063	−0.272 to 0.146	0.64
Treatment duration (months)	0.14	−0.58 to 0.86	0.77

	*RR for radiographic cavity closure*		
Covariate	Coefficient	95% CI	*p*
No. of patients	−0.012	−0.511 to 0.487	0.93
Mean age (years)	0.091	−0.072 to 0.254	0.10
rhuIL-2 dose (10^4^ IU/d)	0.058	−0.122 to 0.238	0.52
rhuIL-2 routes	−0.015	−0.523 to 0.493	0.96
Treatment duration (months)	−0.072	−0.301 to 0.157	0.61

rhuIL-2, recombinant human interleukin-2; RR, risk ratio; CI, confidence interval.

## Data Availability

The data used to support the findings of this study are available from the corresponding author upon request.
